# An *in vitro* investigation of the phytochemical contents of *Marsdenia macrantha* root and its antibacterial activity against selected foodborne pathogens

**DOI:** 10.11604/pamj.2021.40.58.28515

**Published:** 2021-09-24

**Authors:** Nduuvako Christophine Shikwambi, Harris Onywera, Lamech Malagho Mwapagha

**Affiliations:** 1 Department of Natural and Applied Sciences, Faculty of Health and Applied Sciences, Namibia University of Science and Technology, Windhoek, Namibia,; 2 Institute of Infectious Disease and Molecular Medicine, University of Cape Town, Cape Town, South Africa,; 3 Division of Medical Virology, Department of Pathology, Faculty of Health Sciences, University of Cape Town, Cape Town, South Africa

**Keywords:** *Marsdenia macrantha*, *M. macrantha*, phytochemicals, terpenoid, alkaloid, flavonoid, antibacterial

## Abstract

**Introduction:**

Marsdenia macrantha is a crucial source of traditional medicine in Northern Namibia. Its roots are used to treat various health conditions ranging from mouth infections to urinary retention. Despite its medicinal application, there is no known knowledge of its therapeutic properties. Thus, we investigated the phytochemical content and antibacterial activity of M. macrantha.

**Methods:**

M. macrantha root extracts were obtained using three different solvents (distilled water, methanol and acetone) - in the soxhlet and maceration extraction methods. Total phytochemical (terpenoid, alkaloid and/or flavonoid) content was determined by spectrophotometry. Antibacterial activity against common foodborne pathogens (Staphylococcus aureus, Escherichia coli and Salmonella typhi) was determined by both well and disc diffusion method.

**Results:**

we detected the presence of all the tested phytochemicals. Methanol gave the highest percentage yield of extraction (mean: 13.95 ± standard deviation: 0.41%) followed by water (10.92 ± 0.11%) and acetone (6.85 ± 0.23%), F-ratio=326.71 and p<0.0003. The total content determined showed that M. macrantha root extract contained more flavonoids than alkaloids (mg of standard per grams of the dry material). Antibacterial analyses showed inhibitory activity against all the selected pathogens, with the highest inhibition zone against S. typhi (19.7 ± 0.3 mm) - for the acetone-prepared root extract. There were variations in minimum inhibitory concentrations of the extracts prepared by the different solvents.

**Conclusion:**

this is the first study demonstrating the presence of phytochemicals and antibacterial properties of M. macrantha roots. Further studies are needed to isolate and characterize the phytochemicals for antibacterial application.

## Introduction

Ethnomedicinal plants have attracted the attention of scientific and pharmaceutical communities. Such plants are known to contain numerous secondary metabolites, which they use as intrinsic defence mechanisms [1]. These compounds contribute to healing properties against several diseases [2] and have been further exploited in drug discovery and development. Phytochemical class of compounds such as terpenoids, alkaloids and flavonoids are currently used as medications to prevent a variety of diseases and inhibit the growth of various types of cancer cells [3]. Series of studies have reported that these compounds have medicinal properties that include antibacterial, antiviral, anticancer and antimalarial activities [1,4-6].

*Marsdenia macrantha* (*Apocynaceae* family), a shrub natively found in South Tropical Africa and Southern Africa, has been used for generations in Northern Namibia as a medicine to treat various animal and human diseases [7]. It has several African names which include but not limited to “Etanhoka”, “Omufinda-Shilundu” and “Odhingulula” in Oshiwambo language; “Oruzenga” in Otjiherero language; “Dirukwana” in Thimbukushu language (all in Namibia); and “Moraranakwenalerajwe” in Shona (Botswana). In Namibia, it is locally known as “Odhingulula” - due to its creeping nature around other plants. It grows up to 5-10 m with twined branches initially densely short-haired and slightly woody with many lenticels sap clear. The leaves ovate with petiole 10-30 mm long, 20-90 x 15-60 mm rounded or shallowly cordate at base. The seeds are flattened with sessile tuft of hairs at micropylar end [7].

The leaves and flowers of *M. macrantha* have been used to treat snake bites whereas its root powder has been used in the treatment of various ailments and conditions ranging from mouth infections, intestinal problems, constipation, to urinary retention [7]. Phytochemical and pharmacological analyses of flower, leaf and root extracts from medicinal plants have been analysed using different extraction protocols (i.e. methods, solvents and conditions), which have differing extraction performances [1,8-14]. For example, an *in vitro* phytochemical screening analysis of the flower, leaf and root extracts of *Achyranthes aspera* found that methanolic extracts were better sources of phytochemicals (steroids and terpenoids) than chloroformic extracts [11]. To date, the phytochemical content and antibacterial activity of *M. macrantha* remains unknown. Thus, the present study aimed to determine the phytochemical content in the root of *M. macrantha*. Furthermore, its therapeutic properties were examined using common foodborne pathogens, namely *Staphylococcus aureus* (*S. aureus*), *Escherichia coli* (*E. coli*) and *Salmonella typhi* (*S. typhi*) [15], which have contributed to the burden of foodborne illness, including acute gastroenteritis [15].

In Namibia´s capital, Windhoek, the prevalence of foodborne pathogens, specifically that of Shiga toxin-producing *E. coli*, was recently found to be high (42%) in raw beef samples collected from informal and commercial abattoirs [16]. Foodborne pathogens have also been reported to be resistant to known antibiotics [17-19]. Although there have been great advances and ongoing efforts in antibiotic design and development, there is still need to find new antibacterial drugs [17,18,20-22]. Specific phytochemicals (namely 7-hydroxycoumarin (7-HC), indole-3-carbinol (I3C), salicylic acid, and saponin), have been shown to act synergistically with antibiotics (namely ciprofloxacin, tetracycline and erythromycin) targeting *S. aureus* [23]. With this, phytochemicals may be used to repurpose ineffective antibiotics by acting as resistance-modifying agents. We thus, hypothesized that *M. macrantha* harbours phytochemical and antibacterial activity. This study aimed to investigate the phytochemical content and antibacterial activity of *M. macrantha*.

## Methods

**Sample collection:** the roots of *M. macrantha* were collected from the northern part of Namibia following traditional methods that have been used for harvesting. The plant roots were dried for 2 weeks, gridded properly sieved using an analytical sieve shaker (Retsch GmbH, Haan, Germany) and stored for phytochemical analysis.

**Extraction of *M. macrantha* root extract:** to get the root extract of *M. macrantha*, we utilized the Soxhlet extraction method with different solvents (70% methanol, distilled water and 70% acetone). The different solvents were heated along with plant material in a bottom flask and a condenser this minimized the evaporation of solvents (methanol, distilled water and 70% acetone) [10]. Root powder (2.0g) was dissolved in 50.0ml of the different solvents: 70% of methanol, distilled water and 70% acetone. The extraction was then done for 24 hours. Of this time, 21-hour extraction was at room temperature while the other 3 hours comprised heating on a Soxhlet extraction apparatus with a minimum and maximum heating temperature of 90°C and 95°C, respectively. All the extracts were filtered and pre-concentrated using a rotatory evaporator. All the extracts were then stored at 4°C for later analysis. Phytochemical screening was carried out as per a standard protocol described elsewhere [11].

**Qualitative screening of phytochemicals: flavonoids, terpenoids and alkaloids:** in the current study, we evaluated a selected class of phytochemicals. These were: flavonoids, terpenoids and alkaloids. For qualitative screening of flavonoids, 5.0 ml of diluted ammonia (NH_3_) with few drops of concentrated sulphuric acid (H_2_SO_4_) were added to 0.5 ml of the plant extract. An appearance of a yellow colour that disappeared on standing was a positive test for flavonoid. This was confirmed using ferric chloride (FeCl_3_) test. Here, 2.0 ml of the methanolic extract was treated with 2-3 drops of 10% of FeCl_3_solution. A green-blue or violent colouration indicated a positive confirmation phenolic phytochemicals - flavonoids [10,11].

Salkowski´s test was used to detect the presence of terpenoids. Here, 3.0 ml of concentrated H_2_SO_4_ was added to 0.5 ml of the plant extract along with 2.0 ml of chloroform. The appearance a brown ring indicated a positive result [11]. For qualitative screening of alkaloids, plant extracts were dissolved in diluted hydrochloric acid (HCl) and filtered. The filtrates were then treated with Wagner´s reagent: to 0.5 ml of the root extract, 2-3 drops of iodine in potassium iodide (KI) were added. The presence of alkaloids was indicated by the formation of a brown-reddish precipitate [9].

**Quantification of total phytochemicals: flavonoids and alkaloids:** to determine the total flavonoid content in the root extract of *M. macrantha*, 2.0 g of its root powder was macerated in 20.0 ml of different ethanol concentration: 50%, 70% and 99% of ethanol (absolute) each for 4 hours and filtered. A calibration curve generated using six quercetin standards (concentration: 100, 200, 400, 600, 800 and 1,000 µg/ml) was used to calculate the flavonoid content. A mixture of 1.0 ml of the filtrate and 1.0 ml of each of the six standard quercetin solutions was placed in different centrifuge tubes. Thereafter, 4.0 ml of distilled water and 0.3 ml of 5% sodium nitrite (NaNO_2_) solution were added into each tube. After 5 minutes, 0.3 ml of 10% aluminium chloride (AlCl_3_) was added. After 6 minutes, 2.0 ml of 1.0 M sodium hydroxide (NaOH) was added. Finally, the solution was topped-upto 10.0 ml with distilled water [24]. The concentration of total flavonoid content in the test samples was calculated from the calibration plot and expressed as mg quercetin equivalent (QE)/g of dried plant material. All the quantifications were carried out in triplicate and absorbance measured at 510 nm [25,26].

To quantify the total alkaloid content in *M. macrantha*, 2.0 g of its root powder was extracted continuously using a Soxhlet extractor in 20.0 ml of 70% methanol for 24 hours and filtered. The filtrate was evaporated at 45°C to dryness using a rotatory evaporator as described elsewhere [27]. The dried filtrate was mixed with 10.0 mg of hydro-methanolic extract and dissolved in 10.0 ml absolute methanol to give a concentration of 1.0 mg/ml [26]. A calibration curve generated using six caffeine standards (concentration: 100, 200, 400, 600, 800 and 1,000 µg/ml) was used to calculate the alkaloid content in the methanolic extract. To perform this, the root extract (1.0 mg/ml) was dissolved in 2.0 ml of 2.0 M HCl and then filtered. A total of 1.0 ml of this solution was then transferred to a separatory funnel and washed thrice with 10.0 ml of chloroform. The pH of this solution was adjusted to neutral with 0.1 NaOH. In a separating funnel, 1.0 ml of the filtrate was mixed with 5.0 ml of bromo-cresol green (BCG) and 5.0 ml of phosphate buffer. The mixture was extracted using chloroform followed by measuring the absorbance at 470 nm against a blank solution [26,27]. Finally, a line of regression from the caffeine standards was used for estimation of unknown alkaloid content and expressed as caffeine equivalent (CE)/g of dried plant material.

**Antibacterial screening using disc and well diffusion assays and determination of minimum inhibitory concentration (MIC):** to examine if *M. macrantha* root extract had an antibacterial property, we used two agar diffusion methods: i) disc diffusion assay and; ii) well diffusion assay. First, we began by growing selected foodborne pathogens, namely *S. aureus, E. coli* and *S. typhi*, which were maintained on agar slants. Bacterial inoculum was made on the nutrient agar and grown at 37°C for 18-24 hours. Plates were prepared by pouring freshly prepared and adjusted nutrient agar into 10 mm petri dishes. The inoculum was poured directly over the surface of the prepared plates and allowed to solidify.

To perform the disc diffusion assay, sterile 5.0 mm diameter Whatman filter paper discs were applied to the surface of the inoculated plates with sterile forceps. Measured amounts of the crude extracts were inoculated through each disk. The solvents used for extraction (methanol, ethanol, water and acetone) acted as negative controls whereas four antibiotics ciprofloxacin, vancomycin, metronidazole and augmentin were used as positive controls. All plates were incubated at 37°C for 18-24 hours. Zones of inhibition were observed, measured and recorded. This experiment was performed in triplicate. For the well diffusion assay, wells of the same size were punched into the agar containing the bacteria and 100.0 µl of each extract was pipetted into these wells. All plates were incubated at 37°C for 18-24 hours. The diameters of zones of inhibition were observed, measured and recorded. This experiment was performed in triplicate. To determine the MIC of the solvent-prepared extracts, we prepared all the extracts at different concentrations: 100,000 μg/ml, 33,000 μg/ml, 10,890 μg/ml, 3,580 μg/ml, 1,190 μg/ml and 390 μg/ml. Each of these solutions were incubated overnight with separate batches of *S. aureus, E. coli* and *S. typhi*. MIC results were then enumerated using broth dilution.

**Statistical analyses:** after the extraction of *M. macrantha* root extract in duplicate, the percentage (%) yield of the extraction was calculated using the following formula [2]:

$$\%yield=\left(\frac{\text{Weight of the dry concentrated extract}}{\text{Weight of the powdered plant sample used}}\right)\times 100$$

Difference in the mean percentage yield of the three solvent-prepared extracts was computed using ordinary one-way analysis of variance (ANOVA) in GraphPad Prism v6.01 (San Diego, CA, USA). A p-value of <0.05 was chosen as the threshold for statistical significance. Similarly, ordinary one-way ANOVA (with a statistical significance at p<0.05) was used to calculate differences in the mean inhibitory activity as measured by the zone of inhibition (in mm). Two-group comparison between the differences (including that of percentage yield and mean inhibitory activity) using unpaired t test with Welch's correction (assuming a Gaussian distribution), with p<0.05 showing significance.

## Results

**Extraction yield of *M. macrantha* root extract:** all the three solvents (70% methanol, distilled water and 70% acetone) used in the soxhlet extraction of crude extract from the *M. macrantha* root powder resulted in significantly different percentage yields, F-ratio=326.71 and p<0.0003. Methanol yielded the highest percentage yield (mean: 13.95 ± standard deviation: 0.41%) followed by water (10.92 ± 0.11%) and acetone (6.85 ± 0.23%). The results showed significant differences in the means of 70% methanol-prepared extract versus distilled water (p=0.0460), 70% methanol and 70% acetone (p=0.0065) and distilled water versus 70% acetone (p<0.0075).

**Figure 1:**
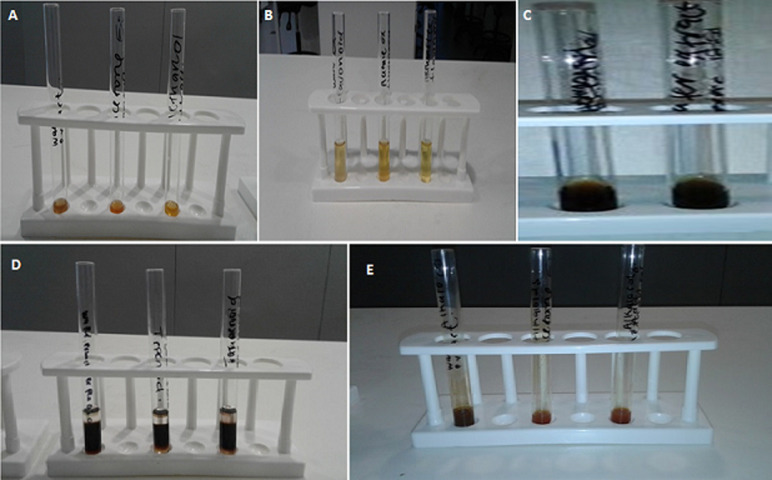
phytochemical screening of phytochemicals: A) orange-coloured crude extracts from roots of *M. macrantha* prior to phytochemical examinations; B) phytochemical screening of flavonoids using ammonium test (an appearance of yellow colour was a positive test for flavonoids); C) phytochemical screening of flavonoids using ferric chloride test (an appearance of green-blue colour was a confirmatory test for flavonoids); D) phytochemical screening of terpenoids using Salkowski’s test (an appearance of a brown ring was a positive test for terpenoids); E) phytochemical screening of alkaloids using Wagner’s test (an appearance of a brown-reddish precipitate was a positive test for alkaloids), all the test tubes (A, B, D and E) except for C (which included 70% acetone prepared extract and 70% methanol prepared extract), were arranged in the following order: water-prepared extract, 70% acetone-prepared extract and 70% methanol-prepared extract

**Figure 2:**
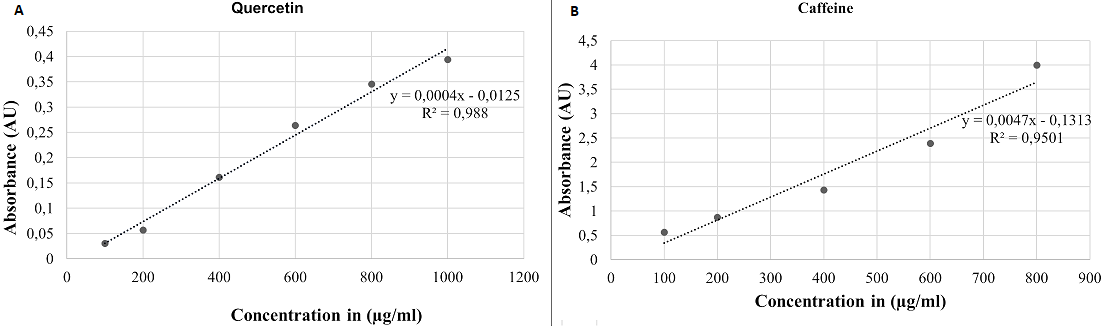
calibration curves for estimating phytochemical content: A) calibration curve generated from six quercetin standards (run in triplicate) whose concentrations (range: 100-1,000) μg/ml) conformed to Beer’s law at 510 nm; the calibration curve had a regression co-efficient (R^2^)=0.988, a slope (m)=0.0004 and y-intercept=0.0125; B) calibration curve generated from five caffeine standards (run in triplicate) whose concentrations (range: 100-800 μg/ml) conformed to Beer’s law at 470 nm, the calibration curve has an R^2^=0.9501, m=0.0047, a y-intercept=0.1313

**Qualitative screening and quantification of phytochemicals: flavonoids, terpenoids and alkaloids:** different colour changes were observed following the addition of various reagents to the orange-coloured *M. macrantha* root extracts, Figure 1. All the phytochemicals were detected in varying amounts in the three solvent-prepared extracts (Table 1). To quantify the phytochemical content, calibration curves generated using quercetin and caffeine standards were used (Figure 2). The flavonoid content of the root powder macerated in different ethanol concentration (mg of QE/g dry plant material) had significantly differing results (F-ratio=519.68, p<0.0001): 99% ethanol (mean: 111.75 ± standard error of mean: 1.36), 70% ethanol (151.32 ± 1.36) and 50% ethanol (191.85 ± 2.36). The concentration of alkaloid content in the methanolic extract was 2.90 ± 0.057 mg of CE/g dry plant material.

**Table 1:** phytochemical screening results of *M. macrantha* roots extract prepared in different solvents

Phytochemicals	Solvents		
	Water	Methanol	Acetone
Terpenoids	+	+	+
Alkaloids	+	+	+
Flavonoids	+	+	+

+: high concentration; +: very high concentration

**Figure 3:**
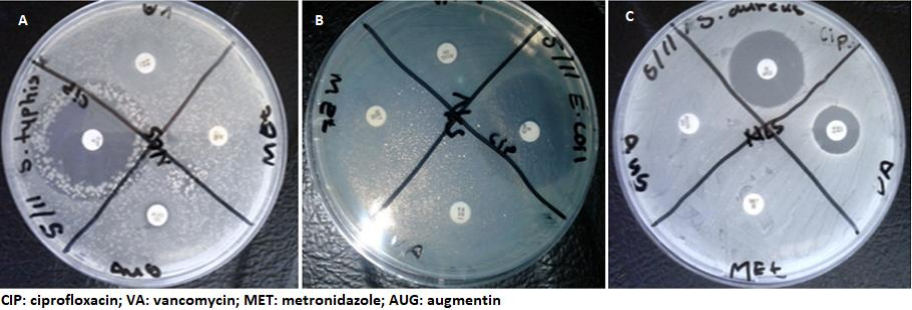
antibacterial activity of selected antibiotics against selected bacterial pathogens using disc diffusion assay: A) inhibitory activity against *S. typhi*; B) inhibitory activity against *E. coli*; C) inhibitory activity against *S. aureus*; all the experiments were performed in triplicate; all the inhibition zones included a 5 mm disc size

**Antibacterial activity of *M. macrantha* roots extracts:** to evaluate the antibacterial activity of *M. macrantha* root extracts, the agar disc and well diffusion methods were carried out. Herein, we measured the diameters of zones of inhibition of *M. macrantha* roots extracts (prepared in different solvents) against selected foodborne pathogens (*S. aureus, E. coli* and *S. typhi*). Known antibiotics (ciprofloxacin, vancomycin, metronidazole and augmentin) were used as positive controls whereas the solvents used for extraction (methanol, ethanol, water and acetone) acted as negative controls. The results for antibacterial activity of the positive controls are summarized in (Table 2 and Figure 3). Of the four antibiotics, ciprofloxacin showed the highest zone of inhibition against the foodborne pathogens. Metronidazole and augmentin showed no inhibitory activity against the pathogens. Whereas, vancomycin did not show any inhibitory activity against *E. coli*.

**Table 2:** antibacterial activity of selected antibiotics against selected bacterial pathogens using disc diffusion assay

Antibiotics	Zones of inhibition (mm)		
	*S. aureus*	*E. coli*	*S. typhi*
Ciprofloxacin (5 μg/disc)	24	30	25
Vancomycin (30 μg/disc)	15	-	19
Metronidazole (30 μg/disc)	-	-	-
Augmentin (30 μg/disc)	-	-	-

-: no inhibition

Antibacterial screening of *M. macrantha* root extracts (prepared by different solvents) indicated that all the solvent-prepared extracts had significantly different antibacterial activities in both the disc and well diffusion methods (F-ratio>20.00, p<0.001) - as shown in Table 3. From the antibacterial screening using the disc diffusion method, we noted that ethanol-prepared extract, followed by that of acetone, had the highest zones of inhibition at 100,000 µg/disc against *S. aureus, E. coli* and *S. typhi*. Methanol- and water-prepared had the lowest zones of inhibition at 100,000 μg/disc and displayed no activity against *S. aureus* and *E. coli*, respectively. Only ethanol- and water-prepared extracts showed zones of inhibition at 1,000 μg/disc, but only against *S. typhi* (F-ratio=117.60, p=0.0004). None of the solvent-prepared extracts showed any zone of inhibition against the bacteria at 500 and 100 μg/disc.

**Table 3:** antibacterial activity of *M. macrantha* roots as measured by disc and well diffusion methods

Antibacterial screening method	Microorganism	Solvent	Extract concentration (μg/disc) (zones of inhibition in mm)							
			100,000 μg/disc	F-ratio&, p-value#	1,000 μg/disc	F-ratio&, p-value#	500 μg/disc	F-ratio&, p-value#	100 μg/disc	F-ratio&, p-value#
Disc diffusion method	*S. aureus*	Methanol	-	414.82, <0.0001	-	N/A	-	N/A	-	N/A
		Ethanol	11.3 ± 0.3		-		-		-	
		Water	6.8 ± 0.1		-		-		-	
		Acetone	9.2 ± 0.1		-		-		-	
	*E. coli*	Methanol	6.8 ± 0.1	397.93, <0.0001	-	N/A	-	N/A	-	N/A
		Ethanol	11.7 ± 0.3		-		-		-	
		Water	-		-		-		-	
		Acetone	8.5 ± 0.2		-		-		-	
	*S. typhi*	Methanol	8.5 ± 0.2	20.96, 0.0004	-	117.60, 0.0004	-	N/A	-	N/A
		Ethanol	10.0 ± 0.5		8.2 ± 0.2		-		-	
		Water	7.8± 0.4		6.8± 0.1		-		-	
		Acetone	10.0 ± 0.5		-		-		-	
Well diffusion method	*S. aureus*	Methanol	-	42.00, 0.0003	-	N/A	-	N/A	-	N/A
		Ethanol	12.0 ± 0.5		-		-		-	
		Water	14.0 ± 0.0		-		-		-	
		Acetone	15.0 ± 0.5		-		-		-	
	*E. coli*	Methanol	10.0 ± 0.5	376.68, <0.0001	-	N/A	-	N/A	-	N/A
		Ethanol	17.3 ± 0.3		-		-		-	
		Water	-		-		-		-	
		Acetone	12.0 ± 0.0		-		-		-	
	*S. typhi*	Methanol	12.3 ± 0.3	468.44, <0.0001	10.7 ± 0.3	124.00, <0.0001	-	N/A	-	N/A
		Ethanol	14.7 ± 0.3		14.3 ± 0.3		-			
		Water	11.3 ± 0.3		11.3 ± 0.3		-		-	
		Acetone	19.7 ± 0.3		-		-		-	

-: no inhibition; N/A: not applicable; & and #: calculations were done using ordinary one-way ANOVA (with a statistical significance at p<0.05)

Further analyses of antibacterial screening using the well diffusion method showed, to a considerable extent, similar results akin to the disc diffusion method (Table 3). Here, acetone-prepared extract exhibited the highest zones of inhibition against bacteria, specifically *S. aureus* and *S. typhi*, at 100,000 μg/disc. Ethanol-prepared extract had the highest zone of inhibition against *E. coli* at 100,000 μg/disc. At 1,000 μg/disc, except for acetone-prepared extract, all the solvents exhibited significantly differing zones of inhibitions, but only against *S. typhi* (F-ratio=124.00, p<0.0001). None of the solvent-prepared extracts showed any zone of inhibition against the bacteria at 500 and 100 μg/disc.

The antibacterial screening results from the disc and well diffusion methods were supplemented with MIC test results (Table 4). Generally, different concentrations of the different solvents were required to inhibit any visible growth of the three bacteria. For instance, the MIC value of water-prepared extract was 10,890 μg/ml for *S. aureus* while that for *S. typhi* was 1,190 μg/ml. Interestingly, the MIC of all the solvents-prepared extracts, except for acetone (33,000 μg/ml), for *S. typhi* was 1,190 μg/ml.

**Table 4:** minimum inhibition concentration of *M. Macrantha* root against selected bacteria

Microorganism	Solvents	Concentration of M. Macrantha root extracts					
		100,000μg/ml	33,000μg/ml	10,890μg/ml	3,580μg/ml	1,190μg/ml	390μg/ml
*S. aureus*	Methanol	-	-	-	α	+	+
	Ethanol	-	α	+	+	+	+
	Water	-	-	α	+	+	+
	Acetone	-	α	+	+	+	+
*E. coli*	Methanol	-	+	+	+	+	+
	Ethanol	-	α	+	+	+	+
	Water	-	-	-	α	+	+
	Acetone	-	+	+	+	+	+
*S. typhi*	Methanol	-	-	-	-	α	+
	Ethanol	-	-	-	-	α	+
	Water	-	-	-	-	α	+
	Acetone	-	α	+	+	+	+

-: no bacterial growth; +: bacterial growth; α: minimum inhibition concentration (MIC) value

## Discussion

This is the first study that shows the antibacterial properties of *M. macrantha* against the growth of bacteria, specifically *S. aureus, E. coli* and *S. typhi*, which are common foodborne pathogens. We successfully extracted medicinal constituents of interest from *M. macrantha* using various solvents, including organic ones [28]. Available literature have found that extraction solvents and methods (processes and/or techniques) result in varying performances, with some solvents and methods outperforming others [8-13]. In our present study, Soxhlet method was used in the sample extraction owing to its ability to extract compounds with low solubility and the fact that it utilizes a little amount of solvent [1,14]. On the other hand, the maceration method was employed for antibacterial activity because of its capability of extracting thermolabile drugs with reduced risk of sample degradation [9]. In our study, the highest yield was obtained with methanol. This observation was similar to previous studies that reported high yields of phytochemicals from *Canarium odontophyllum* leaf and *Achyranthes aspera* flower, leaf and root methanolic extract(s) compared to other solvent extracts, including acetone [11,29,30]. It is well established that successful extraction and determination of phytochemicals is dependent on the nature of the solvent and impacts quantity of the extracted phytochemicals [1,9,12].

Qualitative screening of selected phytochemicals in *M. macrantha* indicated that it contained flavonoids, alkaloids and terpenoids, which occurred at varying concentrations. Flavonoids and alkaloids were detected at high and low concentrations, respectively. This finding mirrored preceding studies, though on other plants - *Kirkia wilmsii* tubers and *Justicia* species, specifically *J. beddomei* and *J. wynaadensis* [27,31]. In our study, the low alkaloid content in the root extracts could be because the root of *M. macrantha* may not be enriched with alkaloids. Our speculation is founded on the premise that a phytochemical screening conducted on *Justicia* found that leaf extracts contained more alkaloids relative to root extracts [27]. Various solvents as well as their concentrations (aqueous degree) may result in different concentration of phytochemicals [14,32]. In our study, the concentration of flavonoids in 50% and 70% ethanol was 1.7- and 1.4-folds, respectively, than in 99% (absolute) ethanol. This is not surprising since water increases the polarity of ethanol, therefore is better at isolating polar phytochemicals like flavonoids from herb matrix [14]. We thus, confirmed the presence of phenolic nature of the flavonoids in *M. macrantha* root extract using ferric chloride test.

Additional experiments to determine the antibacterial properties of the different solvent-prepared extracts showed that, in both disc and well diffusion assays, acetone and ethanol extracts had the highest antibacterial activity against *S. aureus, E. coli* and *S. typhi* as evidenced by large zones of inhibition against these bacteria. However, for the antibacterial activity of the extracts against *S. aureus* in the well diffusion method, acetone and water extracts yielded the highest activity. Interestingly, water and methanol extracts did not show antibacterial activity against *E. coli* and *S. aureus*, respectively. Differences in the antibacterial activity of the solvent extracts could be attributed to differences in extract concentrations, bacterial factors (including structural differences), mechanism of action of the phytochemicals and nature of the solvent. It has been underscored that extracts prepared from organic solvent have more consistent antimicrobial activity compared to water-prepared solvent, which at times my contain water soluble phytochemicals, including flavonoids (mostly anthocyanins), devoid of antimicrobial activity [12]. Use of acetone may result in more and potent antibacterial components against bacteria (this not limited to *S. aureus*) compared to the use water and methanol [12,13].

In terms of mechanism of action, flavonoids binds to adhesins whereas terpenoids disrupt cell membranes and alkaloids intercalate into cell wall [1]. Environment and climatic conditions during plant growth, in this case *M. macrantha*, could have had an impact on the antibacterial efficacy of the phytochemicals [12]. Furthermore, another speculation for the differences in antibacterial activities of the solvent extracts could be due to the existence of other undetected phytochemicals (such as quinones, polyphenols and tannins), compounds/biomolecules (enzymes), and/or microorganisms in the extracts that either potentiated or inhibited the antibacterial activities of the extracts against the selected bacteria [1,2,29,32]. For example, 7-HC and I3C have been reported to affect motility and quorum-sensing of *E. coli* and *S. aureus* [23]. Although previous studies have reported on the pharmacological activities of terpenoids [4,33], alkaloids [5,34,35] and flavonoids [6,35], other factors yet to be determined could also be playing a role in the antibacterial activity of the phytochemicals identified in the root extract of *M. macrantha*.

While our study offers insight into the phytochemical content and antibacterial activity of *M. macrantha*, it had some limitations which are worth highlighting. Firstly, we did not evaluate if all the known phytochemicals were present in *M. macrantha* root extract. Similarly, the specific flavonoids (e.g. chrysin, quercetin and rutin), terpenoids (e.g. caffeic acid, capsaicin, curcumin and trichorabdal A) and alkaloids (e.g. berberine, piperine, palmatine and tetrahydropalmatine) were not characterized. Lastly, the antibacterial screening was limited to only three bacteria.

## Conclusion

This is the first study that shows the antibacterial properties of *M. macrantha* against the growth of bacteria, specifically *S. aureus, E. coli* and *S. typhi*. The root extracts of *M. macrantha* were not active against all the bacteria tested. Further studies should involve screening a wide range of bacteria, viruses, fungi and parasites and individual phytoactive biochemicals with an aim of determining the pharmacological potential and predict toxicity level of *M. macrantha* to microbes and parasites. *M. macrantha* root extract may be an arsenal of phytochemicals with medicinal worth. Therefore, such phytochemicals may be important components of the traditional medicinal system as well as in modern pharmaceuticals. Lastly, our study shows that extractants (solvents) may result in extraction of phytochemicals with different antibacterial properties. This emphasizes on the need to carefully select the extractants in order to maximally extract the phytochemicals.

### What is known about this topic

- Marsdenia macrantha has medicinal value and has been used for generations in Northern Namibia as medicine to treat various animal and human diseases;
- Marsdenia macrantha harbours various phytochemical properties that might be responsible for its medicinal value.

### What this study adds

- Marsdenia macrantha harbours various phytochemical such as flavonoids, alkaloids and terpenoids, which occurred at varying concentrations;
- Marsdenia macrantha has antibacterial properties against the growth of various microorganisms, specifically S. aureus, E. coli and S. typhi.
